# Trust in primary health care and COVID-19 vaccine uptake among Iranian pediatric: a web-based cross-sectional survey

**DOI:** 10.1186/s12887-024-04816-w

**Published:** 2024-05-22

**Authors:** Ehsan Sarbazi, Nazanin Masoudi, Ahmad Mehri, Robab Mehdizadeh Esfanjani, Hosein Azizi, Maryam Soleimanpour, Mahboub Pouraghaei, Hassan Soleimanpour

**Affiliations:** 1 Student Research Committee, Tabriz University of Medical Sciences, Tabriz, Iran; 2 Road Traffic Injury Research Center, Tabriz University of Medical Sciences, Tabriz, Iran; 3 Department of Statistics and Epidemiology, Faculty of Health, Tabriz University of Medical Sciences, Tabriz, Iran; 4 Department of Epidemiology, School of Public Health and Safety, Shahid Beheshti University of Medical Sciences, Tehran, Iran; 5 Neurosciences Research Center, Tabriz University of Medical Sciences, Tabriz, Iran; 6 Women’s Reproductive Health Research Center, Tabriz University of Medical Sciences, Tabriz, Iran; 7 Clinical Research Development Unit of Tabriz Valiasr Hospital, Tabriz University of Medical Sciences, Tabriz, Iran; 8 Emergency and Trauma Care Research Center, Tabriz University of Medical Sciences, Tabriz, Iran; 9 Medical Philosophy and History Research Center, Tabriz University of Medical Sciences, Tabriz, Iran

**Keywords:** COVID-19, Immunization, Vaccine hesitancy, Vaccine acceptance, Epidemiology

## Abstract

**Background:**

Children are the most vulnerable group to diseases. Thus, it’s critical to evaluate parents’ or guardians’ willingness to vaccinate their children. This study aims to investigate the prevalence and predictors of pediatric COVID-19 vaccination in Iran and its relationship with trust in the Primary Health Care (PHC) system.

**Method:**

A cross-sectional online survey of 549 Iranian parents was conducted between January and March 2023. This study collected data from all provinces of Iran using a questionnaire shared on Google Forms and various social media platforms. After considering various background factors, we used a multivariable logistic regression model. This model explored how trust in the PHC system and parent-related and child-related factors were related to parents’ vaccine uptake for their children.

**Results:**

Of 549 parents aged between 19 and 67 years (median = 38 years), 65.2% (358) were female. The prevalence of vaccine uptake among parents was 46.8%. After adjusting for background features, child’s age (adjusted odds ratio [AOR] 0.81, 95% CI 0.71–0.91), vaccine doses (1-dose: AOR 14.72, 95% CI 6.44–33.65, 2-dose: AOR 32.54, 95% CI 15.22–69.57), child’s disease (AOR 5.31, 95% CI 2.19–12.85), and trust in PHC (AOR 1.01, 95% CI 1.00–1.02) were associated with parental uptake of the COVID-19 vaccine.

**Conclusions:**

The findings of this study suggest that the child’s age, vaccine doses received, diseases, and trust in PHC are significant predictors of parental uptake of the COVID-19 vaccine for children in Iran. The results can be used in service planning regarding children’s COVID-19 vaccination.

## Introduction

COVID-19 is a multi-organ response disease with diverse consequences, affecting multiple organ systems and presenting diverse clinical outcomes [1, 2]. Health authorities widely acknowledge the COVID-19 vaccine for children as an effective measure to reduce the burden of COVID-19 infection [3]. As of April 26, 2020, the World Health Organization (WHO) reported seven COVID-19 vaccine candidates in clinical evaluation and 82 in preclinical evaluation [4]. Vaccination plays a pivotal role in safeguarding vulnerable individuals and alleviating the mental, social, economic, and mortality burden of the crisis [5–8].

Large-scale vaccination programs are also necessary to achieve herd immunity, requiring a significant proportion of the population to be vaccinated. The effectiveness of the vaccine is dependent on individual willingness to be vaccinated, which can be influenced by doubts and concerns about vaccine safety and suitability [9, 10]. Moreover, the willingness of individuals to participate in vaccination programs is heavily influenced by the concept of “trust” [5], which can enhance the effectiveness and adherence to treatment [11].

Optimizing vaccination strategies for specific demographic groups, such as children and students, has garnered considerable attention and debate [12]. With the emergence of several new forms of highly transmissible COVID-19 strains, scientists and healthcare professionals recognize the significance of widespread immunization [13, 14].

Research has shown that children are as vulnerable to the virus as adults but are less likely to show or experience severe symptoms [15, 16]. Previous research in Iran encompassed 5,943 children, of whom 13.51% were confirmed via real-time PCR assay [17]. The gender distribution was balanced with a female-to-male ratio of 1:1.3, and the average age was 5.71 years. Approximately 11.2% of the confirmed cases necessitated transfer and admission to the pediatric intensive care unit. Another study in the Golestan province of northern Iran included 91 confirmed cases aged between 0 and 18 years. Of these, 17.5% (16) required ICU hospitalization, and 8.8% (91) resulted in mortality events [17, 18].

The Iranian health authorities granted authorization for the administration of COVID-19 vaccines to children aged between 5 and 11 years. This approval was executed in a phased approach, contingent upon the prior consent of parents [19]. This development was officially reported on January 22, 2022. The decision to vaccinate children ultimately lies with their parents, and their level of trust in vaccines and the primary health care system (PHC) directly impacts their decision to vaccinate [19, 20].

On February 8, 2022, Iran commenced the vaccination of children aged 5 to 12 against COVID-19 [21]. The vaccines administered are Sinopharm and Soberana (PastoCoVac), given in two doses with a minimum of 28 days between doses [21]. As of April 29, 2023, the Ministry of Health in Iran reported that 175 COVID-19 vaccine doses were administered per 100 people. The rate of two-dose vaccination coverage for individuals over 12 in Iran is over 75%, while for children aged 5–12, it is no more than 10% [21]. In December 2021, electronic vaccine certificates were obligatory for employment, military service applicants, and access to universities and schools [21]. However, as of 2024, no COVID-19 testing or vaccination mandates existed for individuals entering Iran [22]. According to a recent systematic assessment, the acceptability of the COVID-19 vaccination among the general population ranged from 23.6% to 97% across nations [23]. According to a global estimate, 57% of parents accept the COVID-19 immunization for their children [24].

Vaccination is a crucial strategy to equip a child’s immune system with the necessary defenses to recognize and neutralize the SARS-CoV-2 virus. Esteemed health institutions such as the American Academy of Pediatrics (AAP) and the Centers for Disease Control and Prevention (CDC) strongly advocate for administering the COVID-19 vaccine to all eligible children and adolescents. However, it should be noted that the vaccination of minors often requires the informed consent of their parents or legal guardians [25].

To make the COVID-19 vaccine more widely available to children, it is critical to understand the parental COVID-19 vaccine uptake of their children and the associated predictors. There is also no information on parents’ attitudes toward vaccinating their children or their trust in the PHC system. Therefore, this study aims to investigate the prevalence and predictors of pediatric COVID-19 vaccination in Iran and its relationship with trust in the PHC system.

## Method

### Study design

This cross-sectional study was conducted from January to March 2023, encompassing various provinces across Iran, with a particular focus on densely populated regions in the west, east, and center of the country. The study utilized an online questionnaire distributed via Google Forms, targeting individuals through university medical science networks, school associations, and parent-student groups on social media platforms such as Telegram, Instagram, and WhatsApp.

### Study population

The study included parents or guardians of children under 18 years old who were aware of their child’s vaccination condition. Participants outside of Iran and those who did not answer more than 50% of the questionnaire items were excluded from the study.

### Trust in Primary Health Care (PHC) scale

Trust in the PHC tool covers 30 statements developed and validated in Iran [26]. The answers to items have five options on a Likert scale of (very little = 1) to (very much = 4). By adding up the scores, the subscales and the total score of trust in PHC are calculated, so the higher the score, the higher the trust is considered. Trust in the PHC tool has two components. The main factor included 25 terms that accounted for 74.1% of the variance, and the specific or optimal task factor included five terms that accounted for 19.2%. Cronbach’s alpha for the whole scale was 98.0. The test–retest reliability for the overall scale using the intra-class correlation coefficient (ICC) was 0.94 [26]. The trust variable is measured on a scale of 0 to 120, with scores falling into three categories: low (0–40), moderate (40–80), and high (80–120).

### Demographic information

A checklist capturing demographic data, including age, gender, education level, and urban or rural residency status, was collected for both the child and the parent completing the questionnaire. Additionally, information regarding the child’s medical history, including conditions such as asthma, chronic kidney disease (CKD), congenital heart disease, respiratory tract infections, thalassemia, congenital anomalies, gastrointestinal infections, and diabetes mellitus, was obtained through parental reports.

### Outcome

The COVID-19 vaccine uptake among parents of children between 5 and 18 years old: ‘Do you intend to vaccinate your children against COVID-19 if the vaccine is available? (Yes/No)' was questioned.

### Sample size

To calculate the sample size, the following formula was used:

$$\mathrm n=\mathrm z^2\mathrm p\left(1-\mathrm p\right)/\mathrm d^2$$

where “n” is the number of samples, “p” is the prevalence [27], considered to be 72%, “d” is precision (0.04), and “Z” is the confidence level value equal to 1.96 for a confidence level of 95% [28]. Finally, the minimum sample size was 480.

### Sampling method and data collection

Convenience sampling was employed to collect data from participants residing in all provinces of Iran. The questionnaire was disseminated via multiple online platforms, including Google Forms, and various social media channels such as Telegram, WhatsApp, LinkedIn, and Facebook. The questionnaire was designed to be easily accessible to participants, with an estimated completion time ranging from 5 to 10 min.

### Statistical data analysis

The data was analyzed using SPSS version 25.0. Due to the non-normal distribution of the data, results were presented as frequencies (percentages), medians, and interquartile ranges. Normality was assessed using the Kolmogorov–Smirnov test and Q-Q plots, and a normal Q-Q plot was determined. Statistical methods such as Chi-square tests, Student’s t-tests, and logistic regression were used for data analysis. Graphs were generated using Graph Pad Prism version 6.0. A *p*-value of less than 0.05 was considered statistically significant.

## Results

### Descriptive analysis for parents

In this study, 549 parents participated, with 65.2% (358) female, and the age range of the parents was between 19 and 67 years (median = 38 years). Of those, 95.1% (522) were married, 49.7% (273) had two children, 74.1% (407) had previous COVID-19, and 28.8% (158) reported COVID-19-related deaths within their families. The education level of about 40% of the parents was a bachelor’s degree (Table 1). Most of the parents (43.4%) received three doses of the COVID-19 vaccine. The prevalence of vaccine uptake among parents was 46.8% (257), with 49.7% among fathers and 45.3% among mothers.

**Table 1:** Demographic characteristics of parents who participated in the study of COVID-19 vaccine uptake among Iranian children between January and March of 2023

Variables	Frequency (%) *n* = 549
Sex	
Male	191 (34.8)
Female	358 (65.2)
Age	38 (42 – 33)^a^
Marital status	
Married	522 (95.1)
Divorced	27 (4.9)
Number of parents’ children	
1	222 (40.4)
2	273 (49.7)
3	54 (9.8)
Job status	
Employed	421 (76.7)
Unemployed	128 (23.3)
History of Parents’ COVID-19 infection	
Yes	407 (74.1)
No	142 (25.9)
Death of relatives due to COVID-19	
Yes	158 (28.8)
No	391 (71.2)
Referred vaccination center	
Rural Health Center	56 (10.2)
Urban Health Center	236 (43)
Public place	110 (20)
Other places	147 (26.8)
Education level	
No formal education	1 (0.2)
Elementary & guidance school	39 (7.1)
Diploma	77 (14)
Associate Degree	73 (13.3)
Bachelor’s degree	223 (40.6)
Master’s degree	100 (18.2)
Clinical specialist	36 (6.6)
Number of vaccine doses	
0	26 (4.7)
1	27 (4.9)
2	174 (31.7)
3	238 (43.4)
4	84 (15.3)
Parents’ acceptance of vaccine uptake	
Yes	257 (46.8)
No	292 (53.2)

^a^Median (Interquartile)

### Descriptive analysis for children

The data indicated that of the children surveyed (Table 2), 60.5% (332) were boys and 39.5% were girls. The age range of the children was between 5 and 18 years, with a mean age of 9 years. The mean age for girls was 8.87 ± 3.98 years, while the mean age for boys was 9.92 ± 3.91 years. Of the children surveyed, 53% had not received any vaccine doses, 11.1% had received one dose, and 35% had received two doses. Additionally, 7.8% (43) of children had a pre-existing medical condition.

**Table 2:** Demographic profile of children of parents who participated in the study of COVID-19 vaccine uptake between January and March of 2023 (*n* = 549)

Variables	Frequency (%)
**Sex**	
Girl	217 (39.5)
Boy	332 (60.5)
**Age**	9 (6–13)*
**Residence**	
City	496 (90.3)
Village	53 (9.7)
**Vaccination doses**	
0	296 (53.9)
1	61 (11.1)
2	192 (35)
**Education level**	
Kindergarten	137 (25)
Elementary 1	190 (34.6)
Elementary 2	137 (25)
High school	85 (15.5)
**Disease**	
Yes	43 (7.8)
No	506 (92.2)

*Median(Interquartile)

### Univariate analysis

No statistically significant difference between fathers and mothers was observed in parent vaccine uptake rates (*P* = 0.316). Moreover, bivariate analysis indicated that parents vaccinated in rural centers had the highest odds of accepting vaccines compared to other vaccination locations. Parents of children who had received one dose of the vaccine were more likely to accept vaccines than parents of unvaccinated children, and parents of children who had received two doses were even more likely to accept vaccines.

### The relationship between trust in PHC and parent’s vaccine uptake

**Fig. 1:**
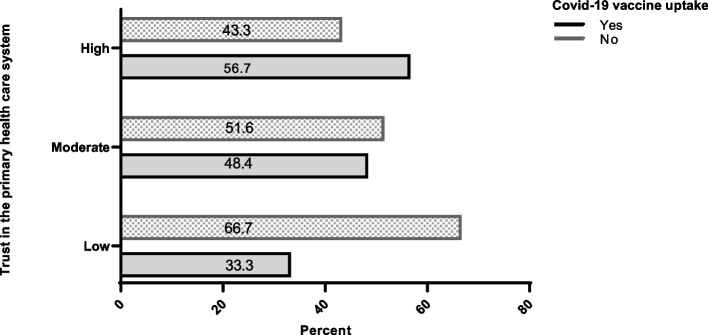
The association between trust in the primary healthcare system and the willingness of parents to uptake vaccine

Trust in the primary healthcare system correlates with parents’ vaccine uptake (*P* < 0.001). A higher score on this questionnaire signifies greater trust in the primary healthcare system. In this study, the mean score obtained from the valid PHC questionnaire was 60.69 ± 28.76, with a minimum score of 0 and a maximum score of 120. The proportion of parents accepting vaccines was 33.3% among those with low trust in the primary healthcare system, 48.4% among those with moderate trust, and 56.7% among those with high trust. In addition, the Chi-square test revealed a significant association between the level of trust in PHC and vaccine uptake among parents. As shown in Fig. 1, as trust in PHC increased, the vaccine uptake rate also improved (*P* < 0.001). Furthermore, the highest vaccine hesitancy was seen in the category with the lowest trust in the primary health center. As shown in Table 3, a significant correlation was found between trust in the PHC and vaccine uptake among parents.

**Table 3:** Comparing the prevalence of COVID-19 vaccine uptake by parents in Iran between January and March of 2023

Variables	Vaccine uptake by parents		*P*-value
	No	Yes	
Sex			
Male	96 (50.3)	95 (49.7)	0.316
Female	196 (54.7)	162 (45.3)	
Age	37.5 (42 – 32)	39 (44.5 – 34)	0.004
Marital status			
Marital	227 (53.1)	245 (46.9)	0.800
Discrete	15 (55.6)	12 (44.4)	
Number of children			
1	126 (56.8)	96 (43.2)	0.229
2	142 (52)	131 (48)	
3	24 (44.4)	30 (55.6)	
Job			
Employed	221 (52.5)	200 (47.5)	0.555
Unemployed	71 (55.5)	57 (44.5)	
Parents’ COVID-19			
No	67 (47.2)	75 (52.8)	0.096
Yes	225 (55.3)	182 (44.7)	
Death related to COVID-19			
No	213 (54.5)	178 (45.5)	0.341
Yes	79 (50)	79 (50)	
Vaccination place			
RHC	28 (50)	28 (50)	< 0.001
CHC	94 (39.8)	142 (60.2)	
Public place	42 (38.2)	68 (61.8)	
Nowhere	128 (87.1)	19 (12.9)	
Child sex			
Girl	118 (54.4)	99 (45.6)	0.651
Boy	174 (52.4)	158 (47.6)	
Child age	9 (12 – 6)	10 (13 – 6)	0.016
Location			
City	262 (52.8)	234 (47.2)	0.600
Village	30 (56.6)	23 (43.4)	
Child COVID-19			
No	173 (54.7)	143 (45.3)	0.394
Yes	119 (51.1)	114 (48.9)	
Child vaccination doses			
0	228 (77)	68 (23)	< 0.001
1	19 (31.1)	42 (68.9)	
2	45 (23.4)	147 (76.6)	
Child education level			
Kindergarten	79 (57.7)	58 (42.3)	0.050
Elementary 1	110 (57.9)	80 (42.1)	
Elementary 2	67 (48.9)	70 (51.1)	
High school	36 (42.4)	49 (57.6)	
Child disease			
No	227 (54.7)	229 (45.3)	0.012
Yes	15 (34.9)	28 (65.1)	
TPHC	55 (76.75 – 34)	67 (87 – 48)	< 0.001

### Multivariable models between demographic factors and parents’ trust in the PHC and COVID-19 vaccine uptake

Multivariable analysis revealed that the child’s age (AOR 0.81, 95% CI 0.71–0.91), child vaccination doses (1-dose: AOR 14.72, 95% CI 6.44–33.65, 2-dose: AOR 32.54, 95% CI 15.22–69.57), child’s disease (AOR 5.31, 95% CI 2.19–12.85), and trust in PHC (AOR 1.01, 95% CI 1.00–1.02) were positive predictors of vaccine uptake among parents after adjusting for parents’ and child’s characteristics (Table 4). After controlling for other variables in the model, parents’ increased trust in PHC was associated with increased vaccine uptake. Additionally, an increase in the child’s age negatively impacted vaccine uptake among parents. The number of vaccine doses the child received significantly predicted vaccine uptake among parents. Additionally, having a pre-existing medical condition increased the odds of vaccine uptake among parents by 5.31 times compared to parents of children without pre-existing conditions. The education level and the number of COVID-19 vaccine doses received by parents were not found to be significant predictors and were, therefore, removed from the model.

**Table 4:** Regression coefficients and adjusted odds ratios for acceptance of vaccination by children’s parents’ predictors derived from multivariable logistic regression analysis

Variables	β Coefficient	*P*-value	Adjusted OR	95% CI	
				Lower	Upper
Age	0.021	0.068	1.021	0.999	1.044
Sex (Ref = Male)					
Female	0.180	0.484	1.197	0.723	1.983
Marital status (Ref = Married)					
Divorced	0.278	0.622	1.320	0.438	3.979
Number of parents children (Ref = 1)					
2	-0.064	0.814	0.938	0.549	1.601
3	-0.023	0.957	0.978	0.432	2.215
Job status (Ref = Employed)					
Unemployed	0.001	0.997	1.001	0.572	1.753
History of Parents’ COVID-19 infection	-0.0532	0.073	0.588	0.329	1.050
Death of relatives due to COVID-19	0.170	0.198	1.185	0.725	1.936
Referred vaccination center (ref = RHC)					
CHC	0.706	0.155	2.027	0.766	5.362
Public place	0.502	0.336	1.653	0.594	4.600
Nowhere	-0.762	0.165	0.467	0.159	1.368
Child’s sex (Ref = Girl)					
Boy	-0.075	0.765	0.927	0.566	1.521
Child’s age	-0.211	**0.001**	0.810	0.719	0.913
Child’s residence (Ref = City)					
Village	-0.346	0.508	0.708	0.254	1.970
Child COVID-19 infection	0.217	0.405	1.243	0.745	2.073
Child vaccination doses (Ref = 0)					
1	2.690	** < 0.001**	14.728	6.445	33.657
2	3.483	** < 0.001**	32.546	15.225	69.572
Child’s education level (Ref = Kindergarten)					
Elementary 1	-0.525	0.199	0.591	0.265	1.319
Elementary 2	-0.604	0.285	0.547	0.181	1.653
High school	-0.440	0.512	0.644	0.173	2.402
Child’s disease^a^ (Ref = no)	1.670	** < 0.001**	5.313	2.196	12.856
Trust in PHC^a^	0.013	**0.002**	1.013	1.005	1.021

^a^*PHC* Primary Health Care system, *OR* Odds Ratio, *CI* Confidence Interval, Child’s disease: asthma, Chronic kidney disease(CKD), congenital heart disease, respiratory tract infection, thalassemia, congenital anomalies, gastrointestinal infections, diabetes mellitus, respectively

## Discussion

This study aims to investigate the parental uptake of the COVID-19 vaccine for children under the age of 18 in Iran and its relationship with trust in the PHC system. Our findings found that factors such as the child’s age, the number of vaccine doses received, diseases, and trust in PHC are all significant predictors of parental COVID-19 vaccine uptake for their children. Notably, increase in trust in PHC was associated with an increase in vaccine uptake by parents.

Trust in PHC is a cornerstone of vaccination uptake and participation [29]. Enhancing trust in health systems directly correlates with increased public confidence in these systems. Consequently, this heightened trust manifests as an increased willingness to receive vaccines and actively engage in vaccination programs [29]. The current study’s findings supported previous research.

The global estimate of parental COVID-19 vaccination uptake for their children was 57% [24]. In a study in China, the prevalence of parents’ acceptance of COVID-19 vaccination for their under-18-year-old children was higher (72.6%) [27], but based on the results of our study, surprisingly, the prevalence of vaccine uptake among parents in Iran was reported to be lower than the global average.

Multiple factors contribute to parental hesitancy in accepting COVID-19 vaccines for their children, including safety [30], effectiveness [30], lack of long-term data [31], risk–benefit analysis [32], trust in the healthcare system [33], fear of unknown consequences [34], and personal beliefs and values [35].

According to the present study’s findings, an underlying disease in children significantly increases the odds of parental vaccine uptake. This suggests that these parents have heightened concerns about their children contracting COVID-19. However, Esposito et al.’s study on parental attitudes towards vaccinating children with illnesses revealed a predominantly negative stance, primarily attributed to apprehensions about potential side effects or worsening of the existing condition [25]. The inconsistency may be attributed to Esposito et al.’s research in the pre-COVID-19 era [25].

Several strategies can be employed to increase parents’ uptake of COVID-19 vaccines for their children. These may be accurate health information, targeted education campaigns [36], building trust in the care system [37], engaging trusted messengers [38], addressing specific concerns [39], sharing success stories and experiences [40], offering vaccine clinics in convenient locations [41], support for decision-making [40], peer influence [42], incentives [24], health-promoting services [43], and continuous monitoring.

Our findings provided an understanding of how to advise vaccination. Parents with younger children and persons with fewer vaccine doses received, the child’s disease status, and trust in PHC must be considered more, as they reported significant predictors of parental uptake of the COVID-19 vaccination.

Given that the parental COVID-19 vaccination uptake for children was relatively low and did not reach the condition of herd immunity, the government and healthcare providers should work to increase parents’ related knowledge and trust in PHC centers.

This study is one of the first of its kind to examine the concept of trust in primary health care and its impact on the willingness of parents to get their children vaccinated against COVID-19 in Iran. In this study, a larger sample size allows for a more representative and diverse group of participants, enhancing the results’ validity. During a pandemic, online data collection methods are more likely to yield valid and accurate data than traditional methods, as they do not require face-to-face interactions or physical contact.

Finally, several limitations need to be considered. One significant limitation of this study was that parents without access to the Internet were not included, which reduced the sample’s representativeness. We could not obtain information regarding people who refused to participate in the study. Parents who refused to participate in the study may have different features than study participants. Moreover, the unavailability of some students’ parents in some parts of the country at the time of data collection could potentially affect selection bias in our study, possibly affecting the generalizability of our findings. Also, reverse causality could be a potential limitation due to the study design.

## Conclusion

In conclusion, factors such as the child’s age, vaccine dosage, disease status, and trust in PHC significantly influence parental uptake of COVID-19 vaccination for children under 18 in Iran. These findings underscore the importance of tailored vaccination promotion strategies to address parental concerns and enhance vaccine acceptance. Service planning efforts regarding children’s COVID-19 immunization should consider these factors to effectively mitigate the impact of the pandemic.

## Data Availability

The datasets used and/or analyzed during the current study are available from the corresponding author upon reasonable request.
